# Reply: expression of carbonic anhydrase IX suggests poor response to therapy in rectal cancer

**DOI:** 10.1038/sj.bjc.6605151

**Published:** 2009-06-30

**Authors:** E Korkeila, K Talvinen, P M Jaakkola, H Minn, K Syrjänen, J Sundström, S Pyrhönen

**Affiliations:** 1 Department of Oncology and Radiotherapy, Turku University Hospital and Turku University, Hämeentie 11, PO Box 52, Turku FIN-20521, Finland; 2 Department of Pathology, University of Turku, Kiinamyllynkatu 10, Turku FIN-20520, Finland; 3 Turku Center for Biotechnology, University of Turku and Åbo Akademi University, Tykistökatu 6, Turku FIN-20521, Finland; 4 Turku PET Centre, PO Box 52, Turku FIN-20521, Finland; 5 Department of Pathology, Turku University Hospital, Kiinamyllynkatu 10, Turku FIN-20520, Finland

Sir,

We were delighted to learn that our article ‘Expression of carbonic anhydrase IX suggests poor outcome in rectal cancer’ (Korkeila *et al*, 2009) had been read with great interest. Span *et al* (2009) pointed out that the results presented in our study only allow the statement that ‘Expression of carbonic anhydrase IX suggests poor response to therapy in rectal cancer’, which we agree upon.

In our study, there were two index patient groups: (i) 75 patients who had received preoperative short-course radiotherapy and (ii) 37 patients who had received long-course radiotherapy with or without chemotherapy. The third group included 54 control patients, none of whom had received any preoperative treatment. In this group there were 34 patients who had not received any postoperative adjuvant treatment (chemotherapy or chemoradiotherapy) either. To test the effect of adjuvant treatment, we compared these 34 patients with all the other patients (*n*=132). There was no statistically significant difference (*P*=0.604) in disease-free survival, and the same was true with disease-specific survival (*P*=0.111) in Kaplan–Meier analysis.

To explore this issue further, we also analysed these 54 control group patients separately, i.e., 20 patients who had received and 34 who had not received any adjuvant chemotherapy. Among those 34 patients who had not received adjuvant therapy, 17 tumours were CA IX-positive and 17 CA IX-negative. Among those 20 who had been treated with adjuvant therapy, 11 tumours were CA IX-positive and 9 were CA IX-negative (*P*=0.722). Among the control group patients who had not received adjuvant therapy, 35% of the tumours had moderate or strong and 15% weak intensity in CA IX staining. Among the patients who had been treated with adjuvant therapy, seven tumours had weak and four tumours had moderate or strong staining intensity (*P*=0.225). There were no statistically significant differences in DFS or DSS between the patients who had or had not received adjuvant therapy.

However, a direct comparison between the groups might be biased by the fact that the patients who had received preoperative therapy had more advanced tumours. According to our practice, adjuvant therapy is given whenever indicated due to a high recurrence risk (node-positive tumours, perforated tumours or tumours that have caused bowel obstruction, tumours with nerve or vessel invasion). All these patients who had received adjuvant therapy had tumours with a higher risk of recurrence.

In conclusion, we agree that ‘Expression of carbonic anhydrase IX suggests poor response to therapy in rectal cancer’.
